# Supplementary material for: Comparative performance of twelve machine learning models in predicting COVID-19 mortality risk in children: a population-based retrospective cohort study in Brazil
Source: PeerJ Comput Sci. 2025 May 28;11:e2916. doi: 10.7717/peerj-cs.2916 (PMC12192853; doi:10.7717/peerj-cs.2916)
Supplement: Supplemental Information 1 [file peerj-cs-11-2916-s001.docx]

**Supplementary Material**


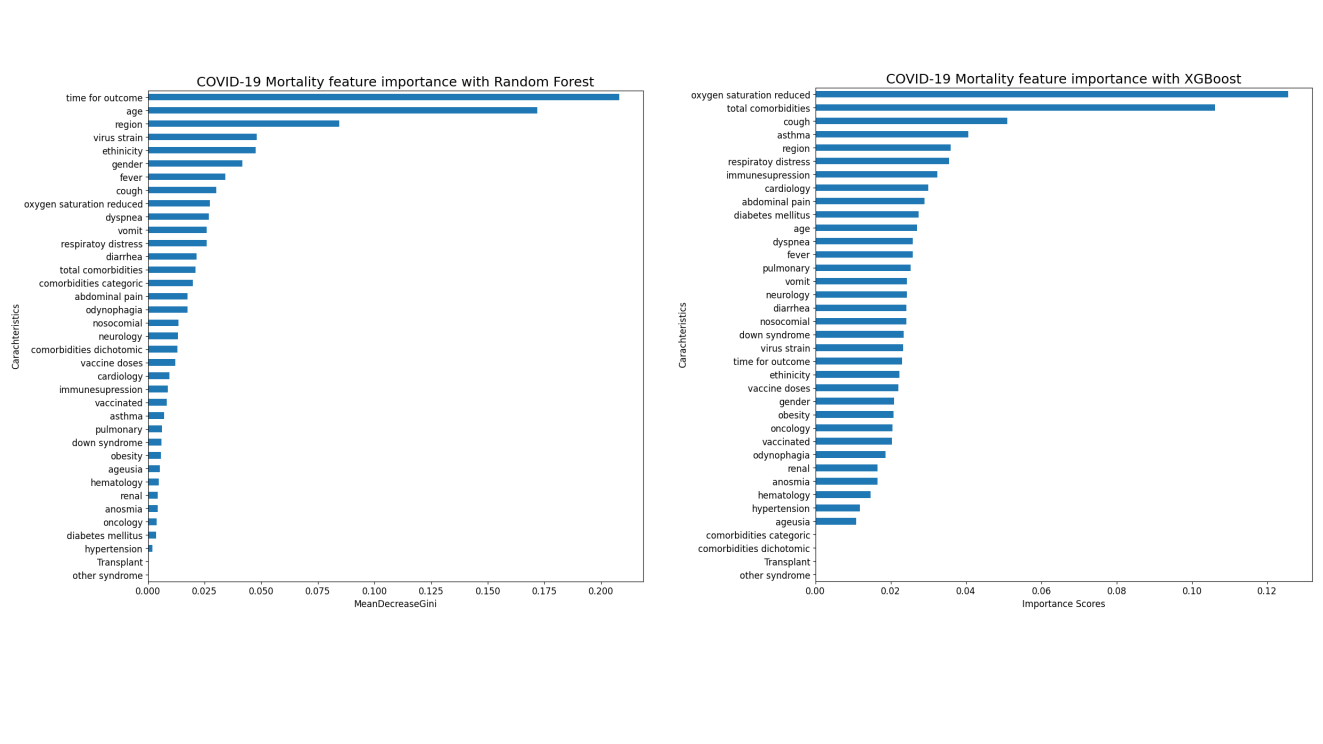


**Supplementary Figure 1** - The importance scores of the predictors were calculated using random forest (a) and XGBoost (b) tests.


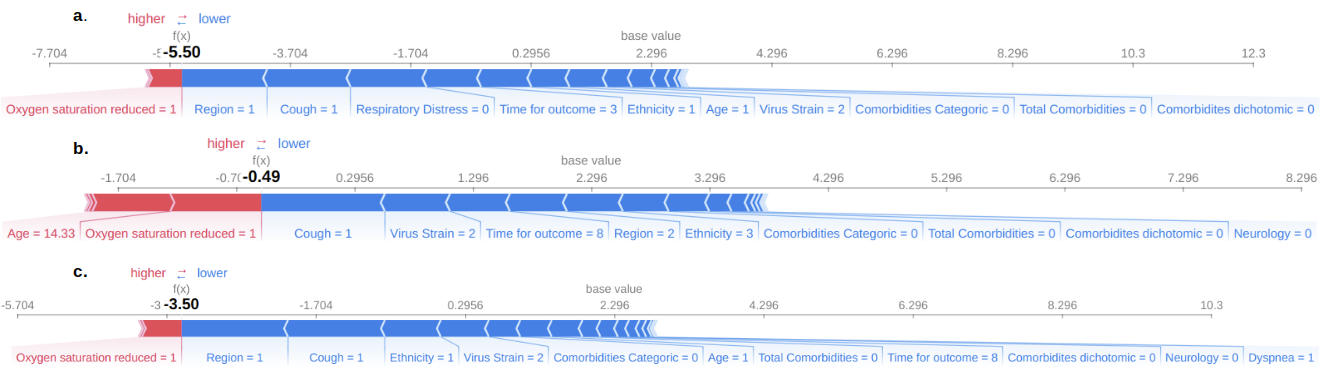


**Supplementary Figure 2** – Force plot of feature contributions to the decision-making process of the model for discharge outcome.

In a force plot for SHAP values, as can be seen in Supplementary Figure 2 (showing specific cases where the model made medical discharge predictions) and Figure 5 (showing specific cases where the model made death predictions), the goal is to illustrate the contributions of individual features to a specific model prediction. Each feature is represented by a horizontal bar, and the length of the bar corresponds to the magnitude of the SHAP value for that feature. The features are arranged horizontally based on their importance to the prediction. We show in each figure three examples of force plots for each class of model prediction (death or discharge).

The plots in Supplementary Figure 2a illustrate the forces for three patients who were discharged from the dataset. Each plot corresponds to an individual patient, and it is evident that reduced oxygen saturation is the most influential feature in the model's decision-making process. However, the force associated with this feature is lower compared to other values, resulting in the classification of the patient into class 0 (medical discharge). It is noteworthy that the region representing the southern region of Brazil had the highest force value, influencing the model's decision to classify the patient as discharged. The absence of respiratory distress, short hospitalization duration, white ethnicity, and the absence of comorbidities were also important factors in the model's decision to classify these patients as discharged. A comparison of Supplementary Figure 2a, 2b, and 2c reveals that lower age values tend to lead the model to classify the patient as discharged, while higher age values, as seen in Supplementary Figure 2b, contribute to the model classifying the patient as deceased. Additionally, variables associated with comorbidities with lower values contribute to the model classifying the patient as discharged.


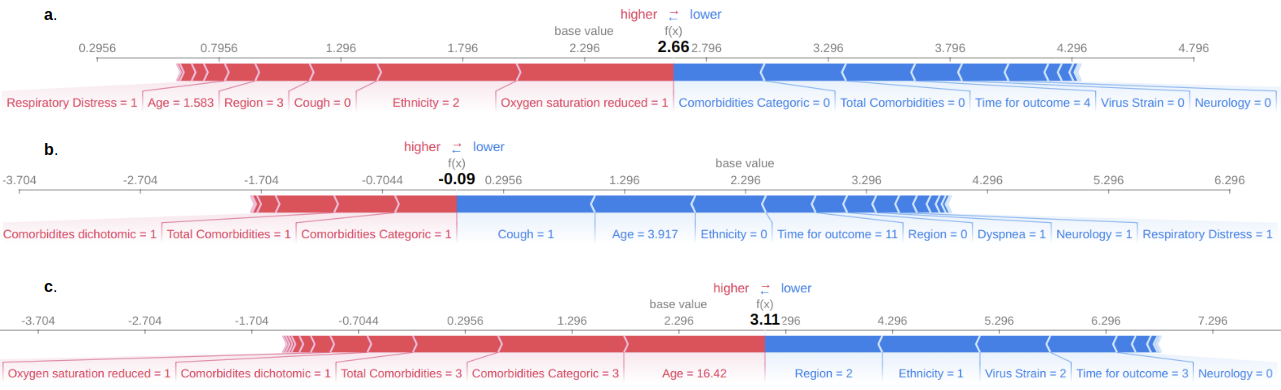


**Suplementary Figure 3** – Force plot of feature contributions to the decision-making process of the model for discharge outcome.

In Supplementary Figure 3a, we observe that a patient presenting reduced oxygen saturation was the primary feature contributing to the model classifying the patient into class one (deceased). Additionally, the model considered an ethnicity value of 2, indicating indigenous, as a variable contributing to an increased prediction value for the higher class, which is deceased. Furthermore, factors such as the northern region of Brazil and reports of respiratory distress contribute to the model classifying the patient as deceased. In Supplementary Figure 3b, the presence of comorbidities emerges as an important factor for the model to classify as deceased. However, solely relying on the presence of comorbidities in patients does not serve as a strong predictor for the model, as the final value of the model's decision function was negative, close to zero. Nevertheless, the model correctly classified this instance as deceased. In Supplementary Figure 3c, besides variables crucial for decision-making regarding mortality, we observe that higher age leads the model to predict the patient as deceased, in contrast to what was shown in Supplementary Figure 2.

**Other studies in the literature that evaluated ML algorithms for predicting deaths in children and adolescents.**

Other studies evaluated ML models for predicting the deaths of children and adolescents from COVID-19. The study conducted by Zhang et al [1]. utilized ML techniques to predict infant mortality rates in the United States based on factors related to birth facility, prenatal care, labor and delivery, and newborn characteristics. The analysis was performed on data from 2016 to 2021, including 116,309 infant deaths among 22,669,736 live births. Among the five ML models compared, XGBoost demonstrated the best predictive performance, achieving an AUC of 93% and an Average Precision (AP) score of 0.55. The study highlighted the significance of utilizing the original imbalanced dataset over balanced datasets created through oversampling techniques, as the former yielded superior predictive outcomes. The validation of the predictive model on data from 2020 to 2021 maintained the performance level, with an AUC of 93% and an AP value of 0.52. The performance of the model during both pre-pandemic (2016–2019) and pandemic periods (2020–2021) shows potential utility in informing strategies to mitigate infant mortality rates.

In the study conducted by Byeon et al. [2], a population-based cross-sectional survey was employed to investigate the impact of the COVID-19 pandemic on the prevalence of obesity among South Korean adolescents. The research utilized categorical boosting, specifically the CatBoost algorithm, to develop a predictive model for adolescent obesity. The model's performance was evaluated using various metrics, and the results indicated that the model achieved an AUC of 68%, with a general accuracy of 82%. The data used in the study encompassed a range of factors including exercise, academic performance, and lifestyle habits, which were analyzed to identify potential risk factors for adolescent obesity. The utilization of the CatBoost algorithm, in conjunction with the evaluation of various performance metrics, underscores the rigorous approach taken to predict vulnerability to obesity in South Korean adolescents post-pandemic.

Gao et al. [3] presents a hybrid approach that combines domain knowledge-based features with data-driven methods to predict pediatric COVID-19 hospitalization and severity. The authors split two cohorts into training, validation, and testing sets by 6:1:3 and used the training set to fit the models, the validation set to determine the hyper-parameters, and the testing set to evaluate the models. The evaluation metrics were AUROC, AUPRC, and Min (Re, Pr). The best model, MedML, achieved a 3% higher AUROC and 4% higher AUPRC on the hospitalization prediction task and a 7% higher AUROC and 14% higher AUPRC on the severity prediction task compared to the best baseline model. The authors used the N3C Data Enclave with Code Workbook and the mini-batch gradient descent to train the models and the batch size was set to 128. The results showed that MedML is generalizable in all nine national geographical regions of the United States and temporally across all consecutive pandemic stages. The authors state that MedML serves as a bridge between clinicians, data engineers, and computer scientists to augment the clinical decision-making process through intuitive knowledge representation, explainable construction, and powerful computation.

Pavliuk et al. [4] developed a ML model for analyzing and predicting the hospitalization numbers of children in the Lviv region during the fourth wave of the COVID-19 pandemic, characterized by the Omicron strain's dominance. The surge in hospitalizations, especially among children, is attributed to their high sociability and low vaccination rates in Ukraine. Utilizing publicly available data, the ML model comprises analysis and prediction components. Pearson correlation coefficient was employed for analyzing hospitalized children's numbers, while short and medium-term predictions utilized neural networks.

The study of Mamlook et al. [5] focuses on evaluating and comparing five well-known ML approaches, including artificial neural network (ANN), random forest (RF), support vector machines (SVM), decision trees (DT), and gradient boosted trees (GBM), to detect COVID-19 in children. The classification performance of each model was assessed using a standard 10-fold cross-validation procedure. The findings reveal that the classification model based on decision trees (CART) outperforms others, achieving 92.5% accuracy for binary classes (positive vs. negative) based on laboratory findings. Important predictors such as Leukocytes, Monocytes, Potassium, and Eosinophils were identified, suggesting their crucial role in COVID-19 detection. The proposed model offers a tool for medical experts to predict COVID-19 in children and validate primary laboratory findings, showcasing the potential of ML methods in facilitating accurate predictions for COVID-19 laboratory outcomes in pediatric cases.

Ma et al. [6] investigate whether clinical symptoms and laboratory results can serve as predictors for the necessity of CT (Computed Tomography) scans in pediatric patients with positive RT-PCR results. Data from 244 pediatric patients were collected, and advanced decision tree-based ML models were employed. The study revealed that age, lymphocyte count, neutrophils, ferritin, and C-reactive protein are crucial indicators for predicting CT outcomes. The developed decision support system demonstrated promising performance, achieving an AUC of 84% with accuracy of 82% and sensitivity of 84%. These findings suggest a reconsideration of CT use in pediatric patients, highlighting the potential non-indispensability of this imaging modality.

Nugawela et al. [7] developed a predictive model for identifying children and young people at a higher risk of experiencing long COVID, defined as having at least one impairing symptom three months after SARS-CoV-2 positive RT-PCR testing. The research utilized data from a nationally matched cohort of SARS-CoV-2 test-positive and test-negative patients aged 11 to 17 years. Predictors considered included SARS-CoV-2 status, demographic factors, quality of life/functioning, physical and mental health, loneliness, and the number of symptoms at testing. The logistic regression model demonstrated an accuracy of 83%, achieving good calibration and discrimination measures.

**References**

1 - Zhang Z, Xiao Q, Luo J. Infant death prediction using machine learning: A population-based retrospective study. Comput Biol Med. 2023 Oct; 165:107423. doi: 10.1016/j.compbiomed.2023.107423. Epub 2023 Sep 1. PMID: 37672926.

2 - Byeon H. Predicting South Korean adolescents vulnerable to obesity after the COVID-19 pandemic using categorical boosting and shapley additive explanation values: A population-based cross-sectional survey. Front Pediatr. 2022 Sep 21; 10:955339. doi: 10.3389/fped.2022.955339. PMID: 36210956; PMCID: PMC9532523.

3 - Gao J, Yang C, Heintz J, Barrows S, Albers E, Stapel M, Warfield S, Cross A, Sun J; N3C consortium. MedML: Fusing medical knowledge and machine learning models for early pediatric COVID-19 hospitalization and severity prediction. iScience. 2022 Sep 16;25(9):104970. doi: 10.1016/j.isci.2022.104970. Epub 2022 Aug 17. PMID: 35992304; PMCID: PMC9384332.

4 - Pavliuk O, Kolesnyk H. Machine-learning method for analyzing and predicting the number of hospitalizations of children during the fourth wave of the COVID-19 pandemic in the Lviv region. J Reliab Intell Environ. 2023;9(1):17-26. doi: 10.1007/s40860-022-00188-z. Epub 2022 Sep 1. PMID: 36065343; PMCID: PMC9434091.

5 - Mamlook, R. A., Al-Mawee, W., Alden, A. Y. Q., Alsheakh, H., & Bzizi, H. (2021). Evaluation of Machine Learning Models to Forecast COVID-19 Relying on Laboratory Outcomes Characteristics in Children. IOP Conference Series: Materials Science and Engineering, 1094(1), 012072. <https://doi.org/10.1088/1757-899X/1094/1/012072>

6 - Ma H, Ye Q, Ding W, Jiang Y, Wang M, Niu Z, Zhou X, Gao Y, Wang C, Menpes-Smith W, Fang EF, Shao J, Xia J, Yang G. Can Clinical Symptoms and Laboratory Results Predict CT Abnormality? Initial Findings Using Novel Machine Learning Techniques in Children With COVID-19 Infections. Front Med (Lausanne). 2021 Jun 14; 8:699984. doi: 10.3389/fmed.2021.699984. PMID: 34195215; PMCID: PMC8236538.

7 - Nugawela, M.D., Stephenson, T., Shafran, R. et al. Predictive model for long COVID in children 3 months after a SARS-CoV-2 PCR test. BMC Med 20, 465 (2022). https://doi.org/10.1186/s12916-022-02664-y
