# Supplementary figures and images for: Comparative performance of twelve machine learning models in predicting COVID-19 mortality risk in children: a population-based retrospective cohort study in Brazil
Source: PeerJ Comput Sci. 2025 May 28;11:e2916. doi: 10.7717/peerj-cs.2916 (PMC12192853; doi:10.7717/peerj-cs.2916)

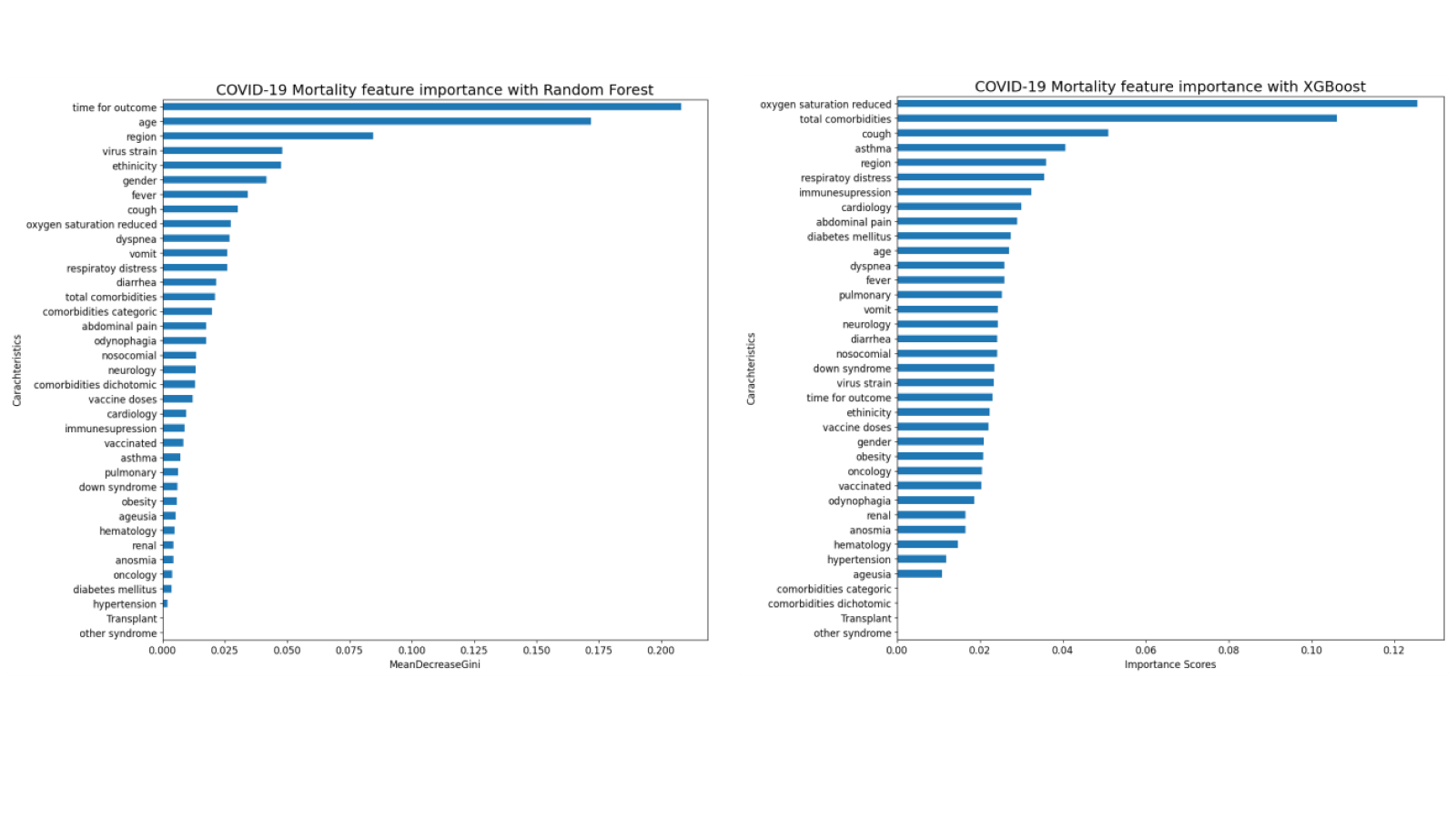

Supplement: Supplemental Information 3 [file peerj-cs-11-2916-s003.png]

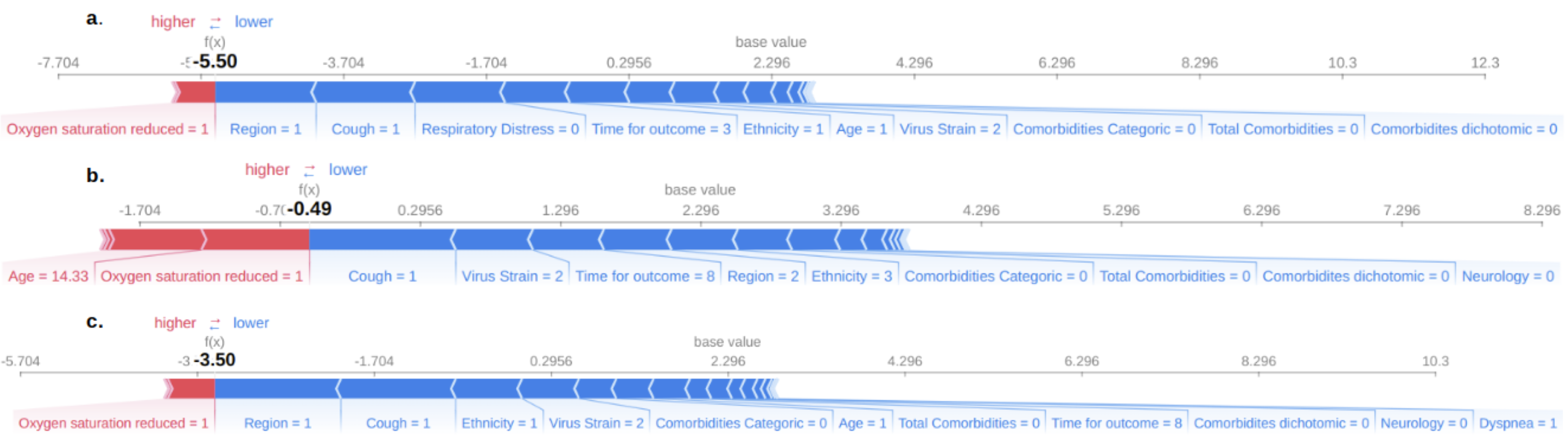

Supplement: Supplemental Information 4 — Features in red are contributing to models deciding to classify patient as dead and blue features contribute to model to classify discharge. In this chart most of the features are contributing to the model to classify discharge. The decision fuction f(x) of the model in all cases is lesser than zero, in this way the model classify patient as discharge. [file peerj-cs-11-2916-s004.png]

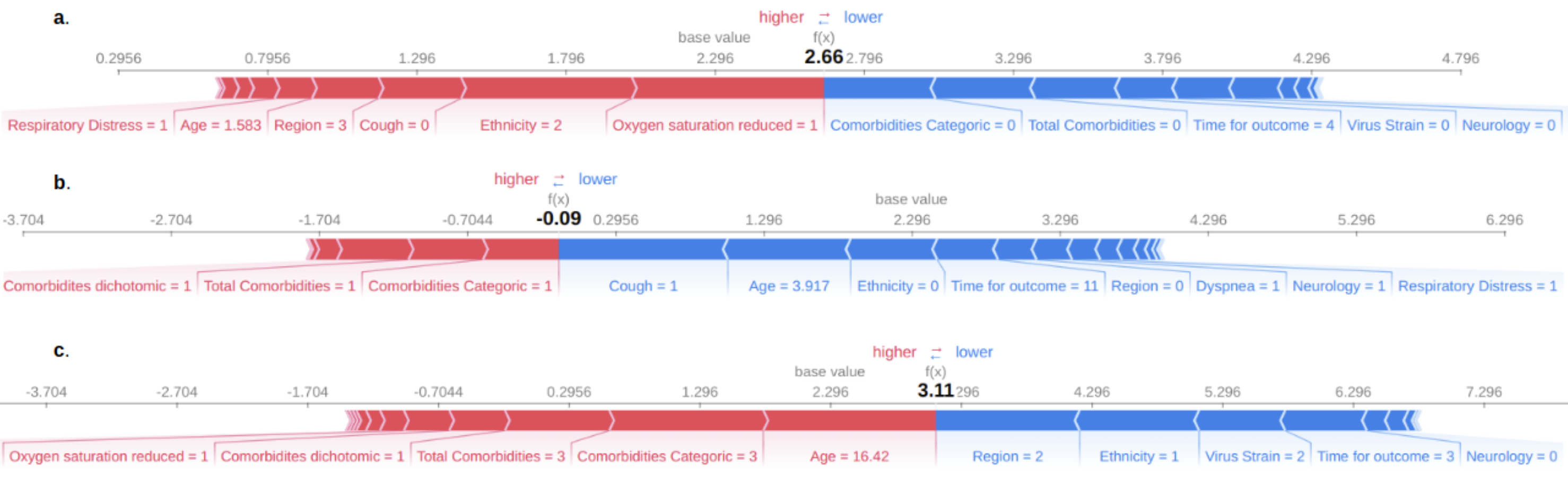

Supplement: Supplemental Information 5 — Features in red are contributing to models deciding to classify patient as dead and blue features contribute to model to classify discharge. In this chart most of the features are contributing to the model to classify death. The decision fuction f(x) of the model in all cases a and c is bigger than zero, in this way the model classify patient as discharge. [file peerj-cs-11-2916-s005.png]
